# Genetic Diversity in the Fusion Gene of Respiratory Syncytial Virus (RSV) Isolated From Iraqi Patients: A First Report

**DOI:** 10.1155/av/8864776

**Published:** 2025-03-28

**Authors:** Mohammed Hussein Wali, Hassan Mohammad Naif, Nur Arzuar Abdul Rahim, Muhammad Amir Yunus

**Affiliations:** ^1^Department of Biomedical Sciences, Advanced Medical and Dental Institute, Universiti Sains Malaysia, Penang, Malaysia; ^2^Department of Molecular and Medical Biotechnology, College of Biotechnology, Al-Nahrain University, Baghdad, Iraq; ^3^Department of Clinical Medicine, Advanced Medical and Dental Institute, Universiti Sains Malaysia, Penang, Malaysia

**Keywords:** fusion glycoprotein, Iraq, PCR, protein modeling, RSV

## Abstract

Molecular evaluation of the respiratory syncytial virus (RSV) genome is one of the common strategies applied to understand the viral pathogenicity and control its spreading. In this study, we carried out molecular evaluation on the targeted fusion (F) gene region in the RSV-positive samples of Iraqi patients during the autumn and winter of 2022/2023. One hundred and fifty patients with lower respiratory tract infections were screened for RSV using reverse transcription-quantitative polymerase chain reaction (RT-qPCR). Sanger sequencing was performed on the RSV-positive samples targeting 1061 nucleotides (from nucleotide 6168 to 7228 within the RSV genome) and 1000 nucleotides (from nucleotide 6122 to 7121 within the RSV genome) of the F gene region for RSV-A and RSV-B, respectively. The results showed some nucleotide changes within the targeted F gene, which were grouped in distinct clade, closely related to isolates from Austria, Argentine, Finland, and France through phylogenetic analysis. In silico protein modeling using the SWISS-MODEL and I-TASSER web tools based on nonsynonymous changes of amino acid sequence showed some good-predicted models that can be utilized for antiviral screening. In summary, the identified nucleotide variations in the F gene could influence vaccine development as the F protein is the primary target for the major antigen of RSV. Molecular surveillance data of RSV local isolates are also essential for studying new genomic changes and enable the prediction of potential new antiviral agents.

## 1. Introduction

Human respiratory syncytial virus (HRSV) is one of the leading causes of pulmonary infections in the lower tract among young children and adults. The virus possesses negative-sense, single-strand nonsegmented RNA, making the viral genome prone to mutations, which further contribute to virus evolution. This virus is highly contagious especially in young babies aged > 2 years old with high capability of re-infection episodes [1–4]. Morbidity rate in RSV cases is relatively high especially in elderly, immune-compromised individuals and infant aged less than 1 year old with figure around 3%–9% globally [5, 6]. RSV infection can lead to a significant number of children being hospitalized with severe bronchiolitis and pneumonia. In 2019, it is estimated that lower respiratory infection cases caused by RSV were around 33 million whereby 3.9 million of cases were categorized as severe and hospitalized patients [7]. Surveillance studies showed that hospitalized pulmonary cases were 1.7% caused by RSV in infants aged less than 6 months and 0.3% in children under the age of 5 years old [1, 8]. It is reported that in each year there are approximately 120,000 deaths among children > 5 years due to RSV-associated hospitalization as a result of ALRI worldwide [2]. Importantly, around 45% of RSV-hospitalized cases and 46% of deaths due to ALRI occur in infants less than 6 years old [9]. The Center for Disease Control (CDC), United States, has reported that in the United States, the annual figure of RSV infections are around 2.1 million cases, 80,000 hospitalized cases in children and 60,000–120,000 cases among elderly group aged ≥ 65 years [10]. A recent global report on the RSV cases revealed that there are around 12 confirmed RSV incidents per 1000 hospitalized children and approximately 1 RSV case per 1000 hospitalized adults [11].

The HRSV virus is classified under the *Orthopneumovirus* genus within the *Paramyxoviridae* family. Its genome consists of a minus single-strand RNA with the size around 15,000 nucleotides that carries 10 genes which encode for 11 proteins [5]. The genes (from 3 to 5 orientations) include nonstructural protein 1 (NS1) gene, nonstructural protein 2 (NS2) gene, nucleoprotein (N) gene, phosphoprotein (P) gene, matrix (M) gene, short hydrophobic protein (SH) gene, glycoprotein (G) gene, fusion protein (F) gene, transcription processivity factor (M2) gene, large polymerase complex (L) gene [4, 12]. Due to the antigenic feature and genetic diversity especially within the G gene region, the HRSV is categorized into two subgroups, RSV-A and RSV-B. The existence of genetic variability in the G attachment gene is responsible for dividing the RSV subgroups into different genotypes [13].

The F protein is expressed on the viral particle surface and it facilitates the viral penetration and fusion to pulmonary cell [14]. Before the viral entry to a host cell, the F protein is found in a prefusion form where it has ultimate structural changes after viral entry. Once the virus enter the host cell, the F protein shifted to postfusion structure which triggers the host immune system [15–17]. The F glycoprotein features six major antigenic sites (Ø, I to V) as identified by a number of studies as potential targets for monoclonal antibody (m-Ab) selection against the RSV [18].

The total length of F gene in RSV-A is 1903 nucleotides located from nucleotide 5619 to nucleotide 7521 in the viral genome, while the length of F gene in the RSV-B is 1900 nucleotides located from nucleotide 5653 to nucleotide 7552 in the viral genome. This gene is very well demonstrated as a conserved region compared to the attachment G glycoprotein gene where the sequence variability is observed in several regions [19]. Because of this feature, the F protein is the common candidate used for vaccine development targeting both RSV subgroups [20].

Recently, two approved vaccines for adults aged ≥ 60 years old are available in the markets [21]. The two approved vaccines are Arexvy produced by GlaxoSmithKline (GSK, United Kingdom) and RSVpre-F produced by Pfizer (Pfizer, USA) [22, 23]. The RSVpre-F has shown good immunization against RSV in pregnant women allowing the production of antibodies against RSV which can be passed to the infant through placenta [24, 25]. Although there is no approved vaccine against RSV in children, there are good improvement in the m-Ab production that can be prescribed for infants who are at high risk of getting infected by the RSV virus [26]. Palivizumab is an effective m-Ab that is usually prescribed as a protective treatment especially for immune-compromised children. Recently, Nirsevimab is a modified m-Ab that has similar action to the Palivizumab with an advantage of having longer half-life and can be taken as a single dose per season [24, 25]. Most of these vaccines and m-Abs target the fusion protein of the RSV, making this part of the virus essential in the immunization against the RSV virus. Importantly, amino acid changes within the fusion protein may affect the vaccine effectiveness by introducing new fusion structure that could alter the immunization process [23]. Changes in the F gene sequence may also improve the RSV fusion mechanism, allowing the virus to escape from the host immune response. The F gene variations could be taken place due to immune-selective pressure with the consideration of a prolonged RSV treatment using m-Ab approach. This enables the virus to alter the antibody binding affinity to the RSV antigenic sites [27]. Additionally, the variations in the fusion gene have been shown to increase the pathogenicity and viral transmission between infected individuals [23, 28].

The emergence of nonsynonymous and synonymous nucleotide changes within the F gene of RSV due to genetic evolutionary over times would contribute to antigenic shift/drift. This phenomenon would impact the development of vaccine and antiviral candidates. Although the rate of genetic changes within the F gene is usually low, a number of studies have highlighted the importance of identifying these changes in order to gain a better understanding of variability profile in the RSV genome [19, 29–31]. Therefore, it is imperative to acquire epidemiological and geographical distribution of the fusion gene variations. The virtual analysis of the RSV viral genome, especially the F gene, has been demonstrated in the literature [5, 32–34]. The in silico analysis of nucleotide changes in the F gene would have a major advantage in searching for conserved regions that could be chosen as candidates for antiviral targets.

In the current work, RSV samples were isolated from hospitalized patients (infants and elderly individuals) in a number of hospitals in Baghdad/Iraq. Upon obtaining a partial nucleotide sequence of RSV-positive samples, a region within the F gene sequence was computationally analyzed to determine the nucleotide changes within the f gene region. Phylogenetic analysis was conducted on the resulted nucleotide sequences of the F gene region and compared with a set of global isolates of RSV in the NCBI Virus database. Based on the nucleotide sequence of the F gene, protein models were constructed to determine the predicted structure for each sample. This research focused on studying the nucleotide changes within the F gene by comparing the results with global isolates of the F gene region, and by studying the effect of possible nucleotide changes on the protein structure of the fusion glycoprotein. Investigating nucleotide changes in this gene is crucial for determination of potential mutational hot spots within the antigenic sites. Therefore, this would further facilitate selection of appropriate targets for vaccines and antiviral agents against RSV.

## 2. Materials and Methods

### 2.1. Sample Size

The proposed sample size is around 100 RSV-positive samples isolated from infected infants and elderly. This sample size was determined by calculation using the *G*∗Power software (http://www.gpower.hhu.de). The calculation was predicted using the point biserial model based on the RSV prevalence of 25% as demonstrated in a recent study in Iraq [35]. The effect size was calculated based on the risk ratio = 0.33 which suggests the sample size to be around 89. The proposed sample size was set to 150 by addition of 15% to the calculated size in order to accommodate possible nonresponses or dropouts.

### 2.2. Sample Collection

One hundred and fifty nasal swab samples were obtained from hospitalized infants and elderly with clinical symptoms of influenza-like illness and acute respiratory tract infections (ARTIs) during autumn and winter of 2022/2023. The samples collection involved three hospitals in Baghdad; Pediatric teaching hospital, AL-Kadhimiya Educational hospital, and Al-Elwya hospital. Specimen collection was done from children and adults based on the common RSV clinical manifestation including chest X-ray and other laboratory tests such as complete blood picture (as advised by medical officers in charge). Nasopharyngeal swab samples were taken (performed by medical officers) within 5–7 days from the onset of the clinical symptoms experienced by the patients after providing a written consent. Other information was recorded from each patient including age, gender, clinical symptoms, existence of recurrent infections, and period of infection. All the swab samples were stored in viral transport media (VTM) and transported to the molecular virology laboratory at AL-Nahrain University where all the samples were stored at 4°C around 72 h before proceeded with RNA extraction procedure.

### 2.3. Screening of RSV-Positive Samples

#### 2.3.1. Primer Design

The variable profile of the viral gnome presents the primary obstacle to the RSV detection approach. A conserved genomic area is utilized in order to attain an accurate viral detection technique. In this instance, a PCR primer set is commonly designed with the (L) gene, which encodes the polymerase enzyme, as the target sequence next to the matrix (M) gene. Well-designed PCR primer sets targeting the L gene have been shown by Todd et al., 2021, with additional modification to match all potential RSV genotypes [36]. These primer sets are specifically made with two probes for the RSV-A and RSV-B genotypes, which enable the detection of both virus types in a single reaction.

All the PCR oligonucleotides employed in this study were analyzed using the NCBI Virus database, in particular, most recent RSV nucleotide database. Several consensuses were chosen to match both RSV subgroups for RT-qPCR-based screening purpose. The identical primer set targeting the polymerase (L) gene from study by Todd et al. 2021 was selected with minor adjustments to the forward primer based on the final sequence alignment (Table 1). This primer set was employed to detect positive RSV samples and differentiate between RSV subgroups.

For amplification of RSV's F gene region, a conserved sequence in the F gene was chosen in both RSV types to design primer sets. Although the F gene is highly conserved in the RSV genome, some nucleotide changes can be observed in the F gene area from different geographical region. Thus, multiple sequence alignment was conducted on several globally available sequences of the F gene in the database for both RSV subtypes. Based on this alignment outcome, a suitable conserved region for primer annealing site was chosen.

#### 2.3.2. Viral Nucleic Acid Extraction

All VTM samples were subjected to RNA extraction and purification by using the Geneaid Viral Nucleic Acid Kit (Geneaid, South Korea) following the manufacturer's instructions. The NanoDrop instrument (ThermoFisher, USA) was then employed to measure the genomic RNA concentration and validate the nucleic acid purity. The extracted RNA samples were stored at - 70°C until the next procedure.

#### 2.3.3. Detection of RSV-Positive Samples

One-step RT-qPCR procedure was performed on RNA samples using the oasig™ lyophilized One-Step RT-qPCR Kit (PrimerDesign, UK). Specific primers sets (targeting the L gene for screening purpose) as described in Table 1 were employed. The RT-qPCR was carried out in a total volume of 25 μL containing 12.5 μL of TaqMan MasterMix, 1.0 μL of forward, reverse primers and probes, and 5 μL of RNA template. The RT-qPCR was conducted using the QuantStudio 5 Real-Time PCR instrument (Applied Biosystems, ThermoFisher Scientific, USA). The cycle condition for the RT-qPCR involved was as follows: holding stage at 94°C for 2 min followed by 40 cycles of denaturation at 94°C for 30 s, annealing at 60°C for 30 s, and extension at 72°C for 30 s. All positive RNA samples with cycle threshold (Ct) value below 40 were selected and stored at - 70°C for further molecular analysis.

### 2.4. cDNA Synthesis

In order to perform a molecular analysis on the F gene of the RSV-positive samples, cDNA was generated from the viral genomic RNA using GoScript™ Reverse Transcription kit (Promega, USA). This procedure was performed on the SimpliAmp Thermal Cycler (Applied Biosystems, ThermoFisher Scientific, USA). Then, purity and concentration of cDNA samples were measured using the NanoDrop instrument (ThermoFisher, USA). cDNA samples with good yield and purity were preserved at - 70°C until the next experiment.

### 2.5. PCR Amplification of the F Gene Region

Specific regions of F gene at location 6168 until 7228 (1061 nucleotides) and 6122 until 7121 (1000 nucleotides) of RSV-A and RSV-B genome, respectively, were used as target for PCR amplification using primer sets as listed in Table 1. The PCR amplification was performed on the RSV-positive samples in a total PCR volume of 50 µL containing 15 μL of MasterMix, 1.5 μL of forward and reverse primers, 5 μL of cDNA template (concentration was set around 100–150 ng/μL). The PCR was carried out using the SimpliAmp Thermal Cycler (Applied Biosystems, ThermoFisher Scientific, USA). The cycling condition involved was as follows: initial denaturation at 94°C for 5 min followed by 40 cycles of denaturation at 94°C for 1 min, annealing at 56°C for 30 s, and extension at 72°C for 30 s. This reaction was completed with a final extension cycle at 72°C for 5 min, and the resulting PCR product was stored at 4°C before further analysis. An electrophoresis on 2% agarose gel was used to examine the PCR amplicons. Using the Bio-Rad XR + Gel Documentation System (Bio-Rad, UK), the targeted PCR amplicon band was found and compared with molecular ladder DM05-01 (Bioland Scientific LLC, USA). Sanger nucleotide sequencing was performed on bands from PCR amplicons that were clear and positive and similar in size to the intended size.

### 2.6. Sanger Sequencing of the F Gene Region

The identical primer sets that were utilized to amplify the RSV F gene region were also used for the Sanger sequencing procedure (see Table 1). Prior to this, the selected PCR amplicons were purified in accordance with the manufacturer's instructions using the ExoSAP-IT Kit (Applied Biosystems, ThermoFisher Scientific, USA). The sequencing procedure was conducted out by Marcogen Company (South Korea), and the resulting sequencing data were subjected to the related bioinformatics analysis. Using MEGA II software and CLC Workbench, each nucleotide sequence that was obtained from the sequencing reaction was examined and cleaned separately. Every sequence that had ambiguous reads at the ends was cut and aligned collectively. Moreover, variable nucleotide/region and gaps were analyzed and stored in a FASTA file format. Nucleotide sequences from some samples were selected and submitted to the NCBI database.

### 2.7. Phylogenetic Analysis

BioEdit Software Version 7.0.5.3 and CLC Workbench software (Qiagen, Germany) were used to analyze and examine all of the targeted RSV F gene nucleotide sequence data that were collected. The NCBI Virus website was employed to acquire a collection of reference sequences of the related RSV-F gene from various geographical locations based on the NCBI database. Using the Multiple Sequence Alignment (MSA) tool in BioEdit and CLC software, a comparison was made between the sequencing data and the NCBI dataset. In the MSA sheet, variations in the nucleotide sequences were found. The CLC workbench software's maximum-likelihood approach was used to build a phylogenetic tree based on the MSA data, allowing for the measurement and assessment of the relatedness, similarities, and differences between the tested samples and NCBI datasets. The tree underwent 100 replicates of bootstrapping in order to assess the dependability of the tree topology.

### 2.8. Amino Acid Sequence Analysis

Using the standard tool included in the CLC workbench program, all of the aligned nucleotide sequences of the targeted RSV F gene site were subjected to codon translation analysis to provide the appropriate amino acid sequences. Among the RSV-infected samples, variations of the deduced amino acid of the RSV F gene were found by comparing the acquired sequences with amino acid sequences from the NCBI datasets. By comparing the obtained F gene amino acids of the local isolates with similar nucleotide sequences from the NCBI database, multiple sequence alignment allows for the detection and observation of the rate of similarities and differences.

### 2.9. Molecular Modeling of Fusion Protein Sequence

#### 2.9.1. F Protein Modeling via SWISS-MODEL Online Tool

Amino acid sequences from each region within the fusion gene were prepared for structure prediction/generation using the SWISS-MODEL tool (https://swissmodel.expasy.org/). First, amino acid sequence was uploaded to the website tool in a FASTA format. Second, the amino acid sequence was validated by the tool before proceeding to the model generation. Then, related protein templates were obtained for each fusion amino acid sequence based on sequence similarity and coverage. Protein structure models were built based on relevant fusion protein templates which were determined by a number of parameters. These parameters are Global Model Quality Estimate (GMQE), quaternary structure quality estimate (QSQE), and identity coverage [37].

After protein model generation, structure assessment was performed to evaluate the predicted model. A few important statistical parameters for each generated model were employed to evaluate each of the model structures. These parameters include GMQE, Qualitative Model Energy Analysis (QMEANDisCo) [38], and QMEAN Z-score analysis [39]. Moreover, a comparison between the target protein sequences and the obtained sequences from the protein database was performed in the SWISS-MODEL to analyze any structure changes within the amino acid chains.

#### 2.9.2. Modeling by I-TASSER Online Tool

The Iterative Threading Assembly Refinement (I-TASSER) is a protein structure and function prediction tool that employs a global template protein library to create prediction of 3D protein structure from any given amino acid sequence (https://zhanggroup.org/I-TASSER/). The tool has direct access to the Protein Data Bank (PDB) library that can be used to build related template and to get information for protein structure and function annotations. Initially, it uses the multiple threading techniques, which is known as local threading meta-server (LOMETS), to identify structural templates from the PDB. Full-length atomic models are then created by iterative template-based fragment assembly simulations. Following this, rethreading of the 3D models through the protein function database (BioLiP), the predicted structures of the F protein were obtained.

### 2.10. Statistical Analysis

The Statistical Package for the Social Sciences (SPSS 20) was used to conduct the statistical evaluation. The chi-square test, which deals with categorical variables, was used to statistically compare the patient's data (gender, age, time of infection, and clinical symptoms). The analysis yielded was deemed statistically significant if the *p* value was less than 0.05. For the phylogenetic trees, each of the generated tree was evaluated by performing 100 replicates of the bootstrapping. Each predicted fusion protein model was statistically evaluated by measuring the QMEAN Z-score as demonstrated above.

## 3. Results

All samples used in the current study were from hospitalized patients with acute lower respiratory infection. These patients primarily had severe influenza-like symptoms, including fever (above 38°C), coughing, wheezing, chest discomfort, breathing problems, pneumonia, and occasionally bronchiolitis. For some cases, particularly those involving newborns, severe respiratory infections, including low oxygen saturation and generalized body weakness, were observed. In addition, a full blood count showed higher lymphocyte counts, indicating possible viral infections.

### 3.1. RSV Incidence Rate and Cohort Characteristics

**Figure 1:**
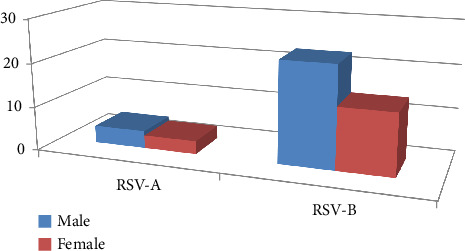
Gender distribution of RSV-positive subtypes A and B among local patients. In both RSV subtypes, the male gender is more susceptible to RSV infections across all age groups.

In this study, the patients' cohort was presented with acute-to-severe respiratory tract infection whereby the nasopharyngeal swab was obtained. Most of the patients in this study cohort were under the age of five, accounting for 105 patients (70%), while 45 patients fall into the adult category. Forty-one individuals were screened for RSV-positive as verified by RT-qPCR analysis. Out of these 41 RSV-positive cases; 34 and seven were linked to RSV-B and RSV-A, respectively. In all age categories, the incidence rate was larger in males as represented by 24 cases, or 58.54% than in females, with 17 cases, or 41.46% (Figure 1). In addition, RSV was found in 9 individuals with the age of over 25 years old and 22 patients with the age under 5 years old. In this study cohort, the incidence rate was greater in the male population whereby 88 (59%) cases were represented by male while 62 (41%) females were among the total cases (*p* value = 0.033).

### 3.2. PCR Amplification

**Figure 2:**
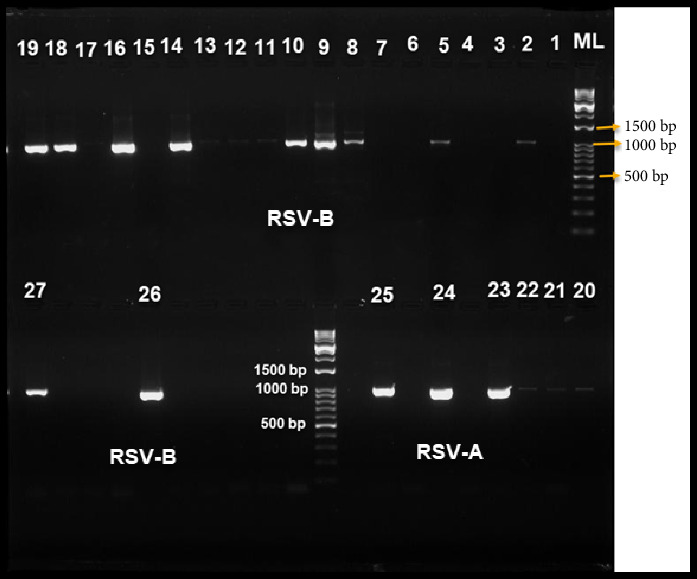
Representative agarose gel image for PCR amplification product (amplicon) of the targeted RSV F gene region that was analyzed by gel electrophoresis. There were amplicons with the specified size bands (1061 bp for RSV-A and 1000 bp for RSV-B). Based on the molecular ladder (ML) by DM05-01 (Bioland Scientific LLC, USA), the size of the F gene bands was determined. The remainder of the examined samples showed similar bands.

The targeted sequence in the F gene was successfully amplified in all 41 RSV-positive samples. As seen in Figure 2, the PCR amplicons for the RSV-positive samples resulted in distinct, observable DNA bands on the agarose gel. The presented bands represent amplicons sized 1061 and 1000 base pair for RSV-A and RSV-B, respectively. After that, the Sanger sequencing technique was used to sequence these PCR amplicons.

### 3.3. Nucleotide and Amino Acid Changes Within the Targeted F Gene Region

**Figure 3:**
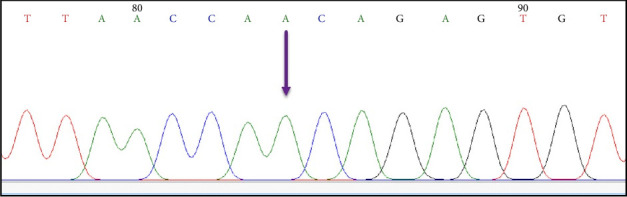
Representative illustration of the chromatogram peak of a single nucleotide substitution at position 56 of the F gene region within the RSV-B genome where guanine (G) is replaced by adenine (A).

The analysis and examination of the sequencing data were done in order to assess the chromatogram peaks for every sample (see Figure 3). To create dependable and unambiguous sequences, terminals for sequences exhibiting unclear signals were excluded from both ends. These sequences and other RSV F gene sequences that were obtained from the NCBI Virus Database were subjected to alignment analysis. Furthermore, each sequence from every sample was examined using the BLAST online tool (https://blast.ncbi.nlm.nih.gov/Blast.cgi). The alignment presented in the BLAST web tool was also used to assess each sample's sequencing data. A few positions within the F gene area showed nucleotide substitutions (Tables 2 and 3). Additionally, the BLAST tool alignment revealed several excellent matches with many nucleotide sequences within the targeted RSV F gene region from different countries, which was examined further using phylogenetic analysis. Importantly, nucleotide variations were observed in the RSV-B samples, whereas no nucleotide changes were detected in the RSV-A samples. Therefore, only nucleotide changes within the RSV-B samples were examined in this study.

A group of partial sequences for the F gene was selected and submitted to the NCBI database. The NCBI submission team looked over and analyzed every sequence area. After the NCBI submission portal verified the sequence, accession numbers were assigned to every sample (OR619507-OR619521).

To demonstrate the relationship between amino acid changes, symptoms, and disease severity, each nonsynonymous amino acid variation was examined based on the number of samples in which the changes were found, as well as the associated disease symptoms and severity. The frequency of samples for each amino acid change was statistically evaluated using the chi-square test (see Table 4).

### 3.4. Phylogenetic Tree of the Tested RSV Isolates Based on Partial F Gene Nucleotide Sequences

**Figure 4:**
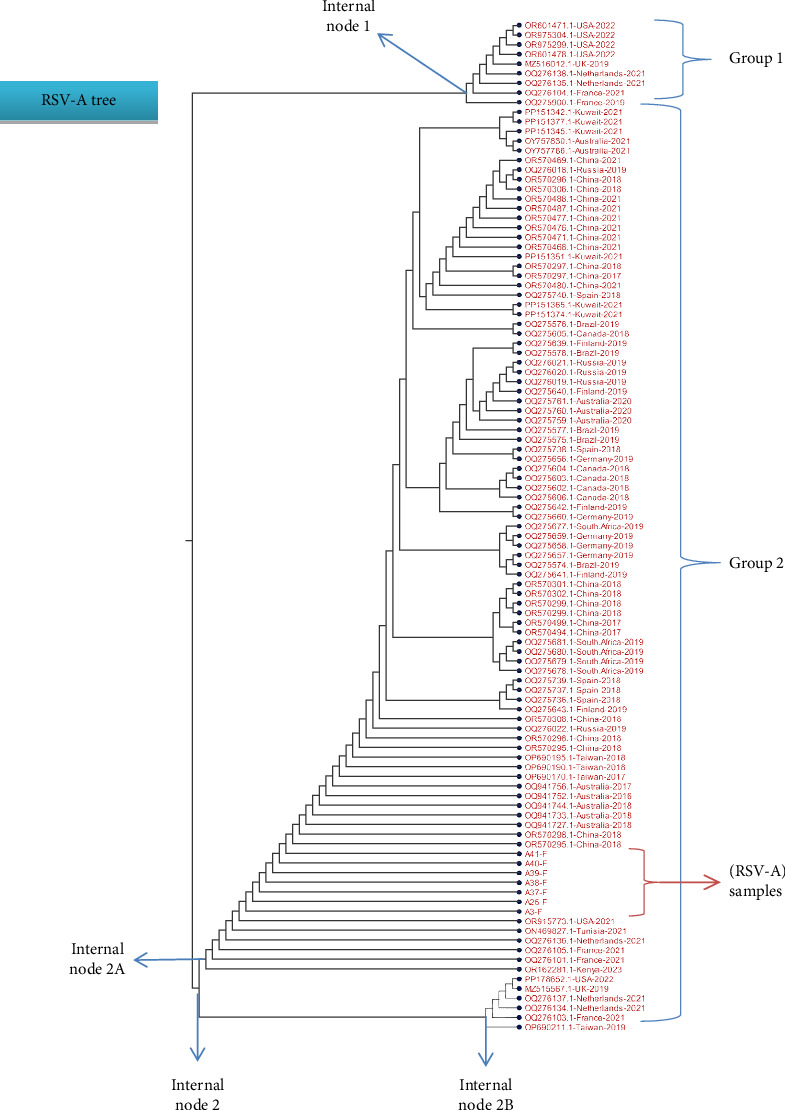
The illustration of phylogenetic tree showing a phylogenetic analysis between the tested RSV-A samples and a selection of NCBI isolates. The tree was constructed using the neighbor-joining algorithm and distance measure by Jukes Cantor method. The tree is divided into two main branches, the top branch, Group 1, include some global isolates, whereas the bottom branch, Group 2, is divided to subbranches that generate internal nodes where our samples are located. All RSV-A samples (red arrow) had emerged from the internal Node 2 which is clustered into two subinternal nodes (2A and 2B). The samples emerged from the internal Node 2A which has a number of sequences from different countries shared similar nucleotide sequences to the tested samples.

**Figure 5:**
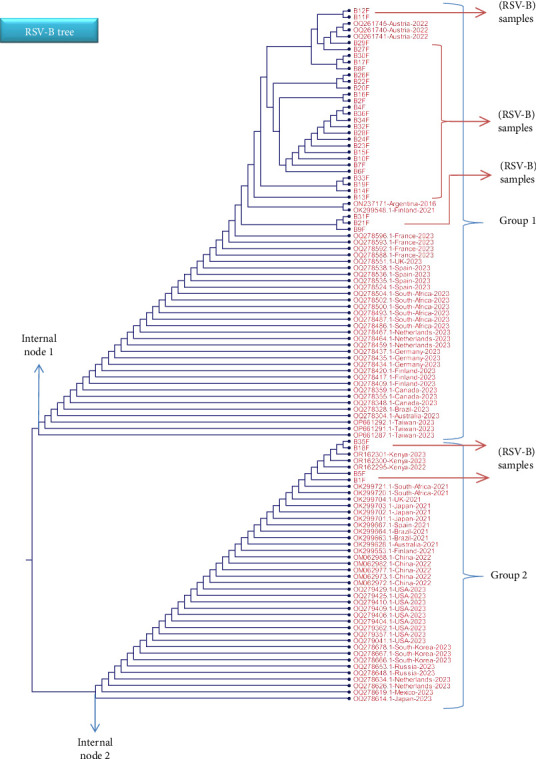
The phylogenetic tree diagram for the F gene region of the RSV-B samples compared with a selection of the NCBI dataset from different countries. The tree was constructed in the Cladogram format where the main branch is divided into two external branches as demonstrated as groups 1 and 2 which in turn different isolates have emerged from. The tree was constructed using the CLC workbench software—Version 8. Most of the samples (red arrows) had emerged from the internal Node 1 where a number of global isolates shared similar sequences to the tested samples. Only four samples had emerged from the internal Node 2 which indicate having some nucleotide variability in the F gene region.

BioEdit Software Version 7.0.5.3 and CLC Workbench software (Qiagen, Germany) were used to analyze and examine all of the specified RSV F gene nucleotide sequences that were obtained. Using the NCBI Virus website, a collection of reference sequences of the related RSV-F gene from various geographical locations was acquired from the NCBI Virus database. Using the Multiple Sequence Alignment (MSA) tool in BioEdit and CLC software, a comparison was made between the sequencing data and the NCBI dataset. In the MSA sheet, changes in the nucleotide sequences were observed. The CLC workbench software's maximum-likelihood approach was used to build a phylogenetic tree based on the MSA data, allowing for the measurement and assessment of the relatedness, similarities, and variations among the tested samples and NCBI datasets. To assess the confidence of the tree topology, 100 replicates of bootstrapping were applied to the tree (Figures 4 and 5).

Details on each variation (usually in a form of single nucleotide substitution) are thoroughly illustrated in the below figures. Each variation is presented in the MSA file that includes some chosen isolates from different countries. For the RSV-A samples, most of the tested samples showed matches with global isolates from Australia, China, Tunisia, the Netherlands, Kuwait, Germany, United Kingdom, and United States, whereas the RSV-B samples depicted high similarity with global isolates from Austria, Argentine, Finland, Spain, and Kenya.

### 3.5. In Silico Modeling of the Targeted F Protein

#### 3.5.1. Modeling by SWISS-MODEL Web Tool

**Figure 6:**
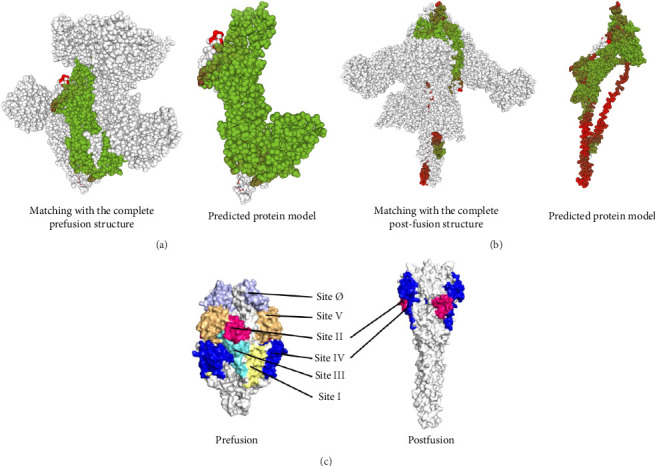
Diagrams of two model structures shown in a space-fill layout. (a) Scheme represents the built model based on the 7uja.1 template that shown some good quality but it has no reliable Z-score value (−3.03). (b) The model structure built based on the 5j3d.1 template with high-quality estimate and good Z-score value. The color scheme of each model structure is represented from green to red based on the local distance difference test (IDDT) score which is described as a superposition-free score that enables the evaluation of each atom in the built model based on their local distance from the chosen template in which the green color indicating high matching quality [44]. (c) The demonstration of the antigenic sites in both pre- and postfusion protein is illustrated in [18].

**Figure 7:**
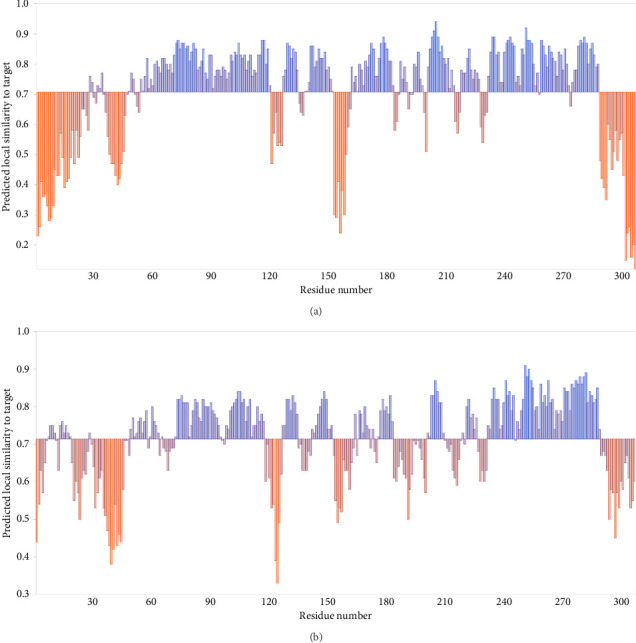
Local quality estimate for the built models allowing the evaluation of similarity to each residue in the model amino acid sequence [40]. (a) Local quality estimate belongs to model that was built using the 7uja.1 template. (b) Quality graph for model generated from the 5j3d.1 template.

A number of structural-predicted F protein models were successfully constructed based on related protein templates from the SWISS-MODEL Template Library (SMTL) (see Figure 6). The results showed seven closely related templates with slight differences in terms of the statistical parameters for each protein structure as demonstrated in Table 5. Each SMLT has specific ID that directly linked to protein library information. The percentage of the matched templates showed high values (above 90%) indicating the degree of close relatedness for each built models with relatively high-quality estimate (see Figure 7). Additionally, the statistical parameters used by SWISS-MODEL, GMQE, and QSQE have shown reliable percentages for all matched templates (the GMQE and QSQE are ranged from 0 to 1; results with values close to one have high expected quality). Most of the chosen templates were structurally determined by X-ray crystallography as it is the recommended structure used in homology modeling. Moreover, the SWISS-MODEL template engine also showed preferred biological ligand for each matched template.

**Figure 8:**
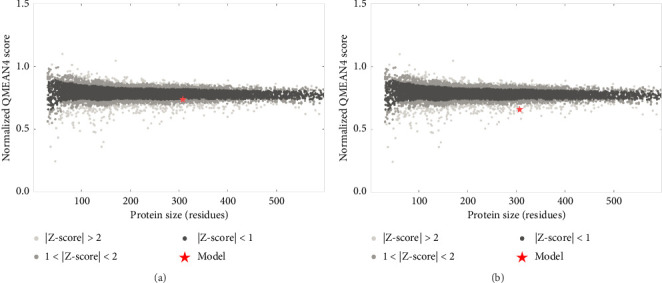
Illustration of the Z-score value for the built models. (a) Protein model with Z-score = −0.90 compared with a set of related protein templates from the SWISS-MODEL template library. The model scoring value is shown within the mean range of the protein templates database indicating a confident scoring value. (b) Protein model with Z-score = −3.03 indicating low-quality model.

The above templates were chosen to build models based on the relatedness with specific protein ID described in the SWISS-MODEL tool. Most of the chosen templates were related to human RSV protein source. Some other F protein sources were chosen to initiate a comparison between the built models.  Table 6 indicates each generated protein model with regard to the quality assessment parameters: GMQE, QMEANDisCo, and QMEAN Zscore. A QMEAN Z-score is computed through eliminating the average normalized QMEAN score and dividing by the standard deviation of the measured distribution [40–43]. The QMEAN Z-score for most samples is below one suggesting a high-quality score to confidentially validate the built models (see Figure 8). The QMEAN Z-score in SWISS-MODEL is a measure of the model's similarity to similar-sized observed structures. Good agreement between the model structure and experimental structures of comparable size is indicated by QMEAN Z-scores below 1 standard deviation [39]. One model was built based on RSV variant template shown Z-score (−3.03), indicating that the model structure is far from the mean score of the template structures.

#### 3.5.2. Molecular Modeling of the Fusion Protein by I-TASSER Web Tool

**Figure 9:**
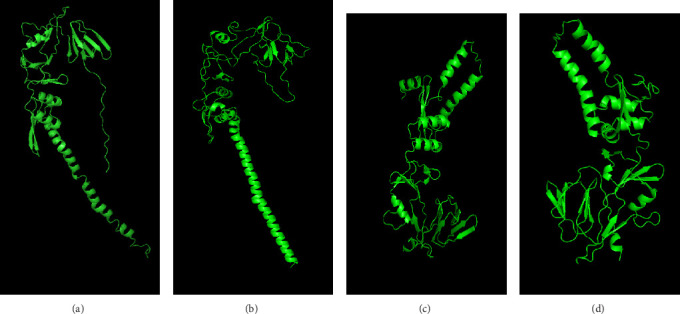
Illustration of the 3D structures of the generated protein models using the I-TASSER web server. Each model was analyzed and processed using the Pymol Molecular Graphic System software (https://pymol.org/). (a) Model numbers 2 and 5. (b) Model Number 4. (c) Model Numbers 3 and 6. (d) Model Number 1 as demonstrated in Table 7.

Several templates from the I-TASSER protein library database were employed to predict the F protein structure employing the uploaded amino acid sequences obtained from this study. Template with high rates of coverage values and identity quality were presented (Table 7). Most of the templates retrieved by the I-TASSER tool depicted reliable score around 0.93 where the identity and coverage are considered reliable when the value is near 1.00 which is calculated based on the complete alignment coverage between the target sequence and the protein template. The constructed models are presented in Figure 9 based on high-quality templates. The normalized Z-score for each template showed different values above 1.00 indicating high-quality score. The normalized Z-score is measured based on the alignment quality score between the target and the database template where it is considered a confident alignment when the normalized Z-score value is more than 1.00 [45–47].

The presented protein structures were chosen based on several I-TASSER statistical criteria that provide quality assessment for each built model. This includes the C-score, Template Modeling (TM-score), and RMSD [45, 47, 48]. The C-score is defined as the value of the model structure quality based on the amino acid alignment with most relevant templates. The C-score value is between −5 and 2 where good-quality model has higher C-score value. The TM-score is calculated based on the assessment of the whole model structure with the global template. One often used metric to compare the similarity of two protein structures is the root-mean-square deviation (RMSD) across comparable atoms of two protein chains. The degree of similarity between the two structures increases with decreasing RMSD. The unit used to measure the RMSD is Angstrom (Å) where the good-quality models have low RMSD score [49, 50].

**Figure 10:**
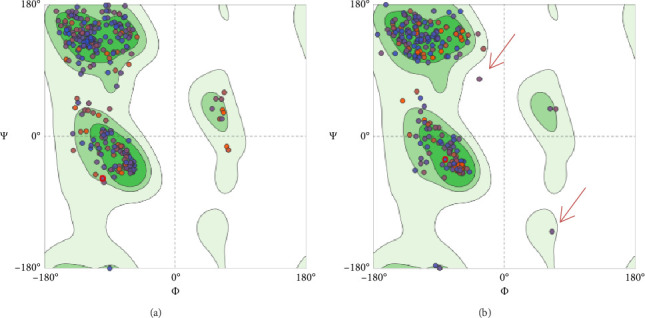
Illustration of the Ramachandran plot for: (a) prefusion model structure showing 96% of the residues in the favored (allowed) region; and (b) postfusion model structure depicting 94.7% of the residues located in the favored region. Red arrows refer to the amino residues located in the disallowed region which only represent 0.65% of the residues.

To assess and validate the stereochemical quality of protein structures for the pre- and postfusion protein models, Ramachandran plots were drawn using the MolProbity Version 4.4 (https://molprobity.biochem.duke.edu). The plots for both models indicated good score as most of the amino acid residues are located at the allowed (favored) region as predicted based on the protein template (Figure 10).

## 4. Discussion

This study highlighted the molecular variations in the F gene nucleotide sequences of the human RSV. To the best of our knowledge, the F gene sequence is considered to be conserved, and that is the reason of choosing this part of the virus as a target for antiviral agents [31, 51]. Any genetic variations in the F gene sequence could lead to alteration in the host immune response and could potentially cause severe respiratory infection. Additionally, any changes in the amino sequence of the fusion protein may cause changes in specific antigenic site leading to block the binding with the antiviral agents [52].

Based on the sequencing results of the F gene region, four nonsynonymous and eight synonymous nucleotide changes were detected in the F gene region of the RSV-B samples. The nonsynonymous alterations led to amino acid variations in the fusion protein of the RSV-positive samples (see Tables 2 and 3). The observed variations are expected to cause some alterations in the immune response against the RSV (Figure 3). This could explain why the virus is able to cause reinfection in the same host within a similar demographic. These moderate amino acid changes are predicted to have no major transformation to the fusion feature of the F protein. However, they may potentially have minor impact to the fusion genetic site especially invading the host immune response. Furthermore, the amino acid alterations in the genetic site of the fusion protein may also impact the antiviral and m-Ab binding process. One of the most used prophylactic m-Ab is Palivizumab where it binds to the site II of the fusion protein [53]. A study conducted in Africa showed that the amino acid changes in a particular genetic site of the fusion gene would have an influence on antibody binding mechanism [52]. The F gene variations were previously reported by Hause et al. [6] who presented some amino acid changes in the antigenic sites of the fusion protein. The study found that the F gene regions of the RSV-B has more amino acid variations than the RSV-A. Similarly, Wang et al. [23] reported that the number of variations in the RSV-B was higher than in the RSV-A subtype in a single study in China. This study highlights the effectiveness of using a particular vaccine that is designed based on the RSV-A strain which may not be working on the RSV-B type. Several mutations were reported in China by Fu et al. [54] who linked these changes with increasing the viral resistance profile against the m-Abs allowing the virus to spread longer during the flu season. This clearly signifies the importance of studying the nucleotide variations within the F gene to enhance our knowledge on the antigenic site changes during a period of time within a particular geographical region.

Changes within the fusion protein could be also linked to severe infections as demonstrated in Table 4. The data indicated that one of the detected amino acid substitutions, specifically at position 19, was highly frequent among the tested patients (88%, *p* value = 0.004) and was associated with severe RSV infections. This could be related to the virus evading the host's immune response, allowing the viral particles to infect more bronchial epithelial cells and cause severe illness with multiple symptoms. Variations in the fusion protein of the RSV had been reported previously to cause higher cytopathological effects in the epithelial cells of the lungs which may lead to severe infection [28]. Additionally, variations at the antigenic sites of the fusion gene would help the virus to escape from the host immunity, leading to multiple infections in the same host during the RSV spreading seasons. Mutations in the antigenic site Ø of the Nirsevimab, the newly produced m-Ab for children, had been reported previously [54]. Although the frequency of nucleotide variations at this site is still low, it is believed that the virus could undergo antigenic drift causing high resistance to the m-Abs [51]. These mutations would also have major effects on the treatment and prevention strategy from the RSV infections. A single study has shown that the mutations in the fusion gene could have an impact on the RSV treatment regime as the virus could have nucleotide variations at its antigenic sits [55]. Inducing the viral mutations in the fusion gene were previously reported in a study showing that despite of the low frequency of these mutations, it is still plausible to have mutant RSV strain as the virus could undergo antigenic shifting and drifting at a specific antigenic site. The occurrence of mutations in the antigenic sites would lead to the generation of RSV-resistant strains that can easily escape from the m-Ab [56].

Furthermore, the detected changes within the RSV fusion protein could be linked to the regional and geographical distribution of the RSV seasonal infection. Several studies have highlighted some mutations within the fusion gene region in different geographical regions [51, 53]. In 2020, Mabilo and his colleague showed an interesting figure of the geographical and epidemiological distribution of the RSV subtypes based on some mutations in the F gene. Their work involved the location of each verified nucleotide changes in the corresponding antigenic site of the fusion protein [52]. Detecting possible mutations within the F gene would be essential for choosing the right target site for antiviral agents.

For the RSV-A tree, the results showed that the seven tested isolates were emerged from the same node, located in Group 2 (see Figure 4), sharing similar clade with global isolates from USA (OR915773.1), Australia (OQ941727.1 and OQ941733), China (OR570295.1 and OR570298.1), Tunisia (ON469827.1), the Netherlands (OQ276136.1). Other global isolates from Kuwait (PP151365.1 and PP151374.1), Brazil (OQ275578.1), Germany (OQ275656.1), United Kingdom (MZ516012.1), and United States (OR601471.1, OR975304.1, OR975299.1 and OR601478.1), were clustered from different nodes, Group 1, indicating that these isolates possess some nucleotide changes in the F gene sequence (Figure 4). The top part of the tree represents a set of global isolates emerged from different clade, meaning they do have high rate of nucleotide changes in their F gene sequence compared with our isolates. In a single study, molecular evaluation of the RSV strains has been studied based on mapping the F gene region for nucleotide changes where they could be linked to specific viral genotype circulating in the population [57, 58].

Regarding the RSV-B phylogenetic tree, the first external branch, presented as Group 1, includes most of the tested sample which shown some good matches with isolates from Austria, Argentine, Finland, and Spain (see Figure 5). The second external branch, Group 2, involves some of the test samples (S1, S5, S18, and S35) and isolates from a number of countries. Importantly, S18 and S35 have revealed high similarity index with isolates from Kenya (OR162295, OR162300, and OR162301). Moreover, S9, S21, and S31 are located near to isolates from Argentina (ON237171) and Finland (OK299548.1). The variability in the F gene is demonstrated in some part of it where some of the nucleotide changes are found in other RSV isolated from different geographical regions. This distribution clearly indicates that F gene region has a high conserved area compared to the G gene region. For this reason, the G gene is the main region used for identification of the RSV subtypes and the further genotypes, whereas the F gene is involved in searching for possible amino acid alteration that could change the structure of specific antigenic site. Different studies demonstrated the importance of mapping the G and F genes for molecular evaluation and epidemiological analysis of the circulating RSV infections [4, 6, 52, 57, 58].

The structural modeling procedures through sophisticated computational methods have facilitated the process of identifying protein variation in a specific RSV protein structure. There are a number of well-designed online tools for producing predicted protein structures that had major assistance in understanding each part of a particular RSV protein [5, 32, 33, 59–61]. The results of the in silico modeling of the fusion glycoprotein have shown some valid structures based on the amino acid sequences of the tested RSV isolates. The SWISS-MODEL has revealed a few matched templates related to the human RSV fusion protein. By comparing the statistical parameters, such as GMQE, QSQE, and identity percentage, models were generated from each chosen template (Table 5). Most of the built models were constructed based on mainly three fusion protein templates: 7uja.1, 5j3d.1, and 7uj3.1. The predicted models (see Figure 6) were closely related to the postfusion protein form with good-quality scores based on the tested parameters: QMEANDisCo and QMEAN Z-score (see Table 6 and Figures 7 and 8).

For model structure evaluation, I-TASSER server was also employed to build models using the amino acid sequences of the tested samples. This server showed some good templates related to the post- and prefusion forms where the postform templates have high-quality matching scores (Table 7). The produced models were also verified based on the I-TASSER statistical parameters as demonstrated in Table 8. Two similar model structures were built by each server that are related to the postfusion form where they have similar structural presentation in both SWISS-MODEL and I-TASSER servers (Figures 6 and 9).

Both protein models were evaluated by employing the Ramachandran plot which allows the evaluation and validation of the stereochemical quality of protein structures, particularly in computer modeling and structural biology (Figure 10). Based on established structural preferences, Ramachandran plot is employed to examine and assess the quality of protein backbone dihedral angles (phi ϕ and psi Ψ). This method is effective and practical as it uses just two variables, the φ and ψ angles to examine and assess three-dimensional protein structures [62–64]. The results for Ramachandran plots depicted good stereochemical quality for both model structures where most of the amino acid residues located in the upper right area (the allowed region of the Ramachandran plot). Only 0.65% of the residues are located in the disallowed region for the postfusion protein model (red arrows in Figure 10). Furthermore, the generated protein models were assessed using several evaluation parameters such as GMQE, QMEANDisCo, and QMEAN Z-score for the models predicted by the SWISS-MODEL tool (Table 6), and C-score, TM-score, and RMSD that were performed by the I-TASSER tool (Table 8) as thoroughly explained in the results.

The presented model structures have a potential role in identifying any changes in the antigenic sites of the fusion protein which could alter the host immune response or may interfere with the antiviral compound's interactions with the RSV fusion residue. By employing the modeling methods in each local RSV isolate, specific antiviral compounds (ligand) could be suggested to directly target specific antigenic site and prevent the RSV spreading within a certain geographical area. The in silico molecular docking, which targets the fusion protein forms (i.e. pre- and postfusion forms), had been addressed very well in several research studies. The importance of these computational methods lies in finding potential antiviral agents targeting particular viral proteins [5, 18, 33, 59, 65]. Although it would be beneficial to perform molecular docking for certain ligands with the built models, the docking process requires models that represent the whole fusion glycoprotein structure. It is clearly shown that performing protein modeling on variable sequences of the F protein would improve our knowledge on the possible outcome of viral mutations. Studying the predicted protein model would definitely enhance the surveillance data on the fusion protein. There are some limitations in this study. Firstly, covering the full length of the F gene would provide further knowledge on other possible nucleotide changes allowing more precise mapping of this gene. Secondly, increasing the sample size would identify the precise frequency of each amino acid changes within the fusion protein among the infected population. Lastly, this work focused on RSV samples isolated from Baghdad/Iraq; thus, the study is limited to one geographical area. Obtaining RSV samples from other regions of Iraq would give a wide insight on the RSV distribution and nucleotide variations in the F gene area in the region.

## 5. Conclusion

In summary, gathering monitoring data on RSV infections throughout the year or season is critical to comprehending the behavior of the virus and ultimately halting its transmission. According to the study's results, the percentage of RSV-positive is still rather high (27%) and the remaining instances may be caused by other respiratory viruses or bacteria. The variation profile of the fusion gene sequence is still low compared to the G gene as predicted and shown in the literature. The detected amino acid changes may cause structural changes in the fusion protein which could alter the binding with certain antiviral agents. Our study presented the first epidemiological report on the RSV cases in Iraq based on the fusion gene sequence. A selection of the nucleotide sequence of the F gene was successfully uploaded and verified by the NSBI website. The phylogenetic analysis revealed that the F gene region of RSV can be employed to show the relatedness with the global RSV isolates. However, defining the RSV genotypes by mapping the F gene would not be feasible as most of the F gene areas are conserved regions. Mutations within the F gene regions could be linked to geographical distribution of the RSV cases where certain nucleotide changes could be triggered by regional factors. The location of amino acid changes in this gene would be essential in defining any possible changes in its antigenic sites. In terms of vaccine development, studying these changes would be essential in finding suitable targets for vaccine and m-Ab. Moreover, predicting the fusion protein structure could be employed in the production of specific vaccine based on a certain area of the fusion protein. Based on the current findings, performing protein modeling on the obtained nucleotide sequence of the RSV F gene is a novel approach for getting a predict protein structure that could be used in the vaccine development. It is recommended to routinely sequence the F gene region of the RSV, which could save time and efforts in finding the best antiviral agents. In addition, performing whole genome sequencing on the RSV genome would generate valuable data for predicting protein models based on the observed nucleotide variations. The *In silico* protein modeling and molecular docking would give a great promise to identify the best vaccine candidates and antiviral compounds against the circulating RSV genotypes within a certain region.

## Figures and Tables

**Table 1:** Details of PCR oligonucleotides used for RSV detection targeting the L and F genes. The sequence, location, and product length of primers were based on the RSV reference sequences with accession numbers NC_038235.1 and NC_001781.1 for RSV-A and RSV-B, respectively.

No	Primer name	Sequence	Gene name	Location	Tm	Product length
1	Real-time PCR	F:AATACAGMMAARTCYAAYCAACTTTAYA	L	**13,850**	**59.8**	**94**
		R: GCCAAGGAAGCATGCARTARA		**13,943**	**57.7**	
		RSV-A-Probe: CYTTARTRCACAATAGCA	L	**13,899**		
		RSV-B-Probe: GACATCYTTAGTAAGGAAYAGTG		**13,905**		

4	RSV-A-F	F: GCTCTACTATCCACAAACAAGG	F	**6168**	**56.5**	**1061**
		R: GTGGTGGATTTACCAGCATTTAC		**7228**	**58**	

5	RSV-B-F	F: GCTGTATCCAAAGTTCTACACC	F	**6122**	**56.7**	**1000**
		R: CAGAAGGAAACACTAGAGGGTC		**7121**	**57.8**	

*Note:* The bold values in the location column refer to the exact location of each targeted fragments of the L and F gene regions within the RSV genome. Tm refer to the melting temperature for each primer set. The fragment size for each primer set is demonstrated in the product length column. These values are essential in the real-time PCR and conventional PCR experiments which was designed to amplify the F gene region of the RSV.

**Table 2:** Details of the single-nucleotide variations detected within the tested area of the F gene region in the RSV-B samples. The nucleotides labeled with red represent the substitution in each codon which in turn may cause amino acid changes (nonsynonymous mutation) or no substitutions occur (synonymous mutation). The type of each variation in the last column is written based on the detected variations in each codon.

NO.	Position on the F gene	Variation	Codon changes	Amino acid substitution	Note
1	**9**	A > G	ACA > ACG	T > T	Synonymous mutation
2	**56**	G > A	AGC > AAC	S > N	Nonsynonymous mutation
3	**119**	G > A	AGT > AAT	S > N	Nonsynonymous mutation
4	**414**	T > C	ATT > ATC	I > I	Synonymous mutation
5	**421**	C > T	CCT > TCT	P > S	Nonsynonymous mutation
6	**433**	T > C	TTA > CTA	L > L	Synonymous mutation
7	**546**	A > G	AAA > AAG	K > k	Synonymous mutation
8	**652**	T > C	TCC > CCC	S > P	Nonsynonymous mutation
9	**708**	T > C	ATT > ATC	I > I	Synonymous mutation
10	**762**	C > T	TCC > TCT	S > S	Synonymous mutation
11	**801**	T > A (B2&B16)	GGT > GGA	G > G	Synonymous mutation
		T > C (7 samples)	GGT > GGC	G > G	Synonymous mutation

*Note:* These values are essential in underlining the exact position of each single nucleotide variations within the F gene region of the RSV genome.

**Table 3:** Illustration of the amino acid changes in the fusion protein region among the RSV-B isolates from the participated patients. Similar to the last table, nucleotides colored red denote alterations that happened at a specific location according to alignment analysis performed using a collection of NCBI RSV isolates from the amino acid database.

NO	Position	Codon in NCBI isolate	Amino acid symbol (name)	Codon in samples	Amino acid symbol (name)	Note
1	**19**	AGC	S (Serine)	AAC	N (Asparagine)	This was detected in most of the samples and in isolates from Austria
2	**40**	AGT	S (Serine)	AAT	N (Asparagine)	This variation was seen in four samples and isolates from Austria
3	**141**	CCT	P (Proline)	TCT	S (Serine)	This was found in sample No. 4 only
4	**218**	TCC	S (Serine)	CCC	P (Proline)	This substitution was observed in four samples and in the same isolates from Austria

*Note:* These values give the exact location of the detected amino acid variations within the fusion protein sequence.

**Table 4:** The relationship of the detected amino acid variations among adults and children with the common symptoms and disease severity.

NO	Position	Amino acid variation	Number of samples	Symptoms	Severity	Number (frequency %)		*p* value
						Adults (total = 8)	Children (total = 26)	
1	**19**	S > N	**25**	Fever, cough and breathing difficulties, wheezing, and pneumonia	Moderate to high	**2 (25%)**	**23 (88.46%)**	**0.004**
2	**40**	S > N	**4**	Fever, wheezing and severe cough	Moderate	**0**	**4 (15.38%)**	**0.273**
3	**141**	P > S	**1**	Fever and breathing difficulties	High	**0**	**1 (3.84%)**	**0.573**
4	**218**	S > P	**4**	Fever, wheezing, and pneumonia	High	**1 (12.5%)**	**3 (11.53%)**	**0.941**

*Note:* These values represent the number and frequency of the detected amino acid variations within the fusion protein among the adults and children patients. The p values were calculated based on the obtained frequencies for each amino acid variation. These values are essential in understanding the relationship between the detected amino acid variations and the disease symptoms.

**Table 5:** Summary of suggested templates retrieved in the SWISS-MODEL for each protein sequence.

No	SMTL ID	Template name	GMQE	QSQE	Identity (%)	Method	Ligand
1	7uja.1	A RSV variant (construct pXCS847A) F1	0.90	0.69	93.49	EM	NAG⁣^∗^
2	5j3d.1	F fusion glycoprotein—crystal structure of human fab 14N4 in complex with postfusion RSV F	0.87	0.69	93.18	X-ray, 4.1A	None
3	5j3d.1	F fusion glycoprotein F0	0.87	0.70	92.53	X-ray, 4.1A	None
4	5j3d.1	F fusion glycoprotein F0	0.87	0.69	92.86	X-ray, 4.1A	None
5	7uj3.1	B RSV variant (construct pXCS847A) F1	0.90		93.16	X-ray, 4.3 Å	NAG
6	7uja.1	A RSV variant (construct pXCS847A) F1	0.90	0.69	93.16	EM	NAG
7	5j3d.1	F fusion glycoprotein F0	0.87	0.67	92.83	X-ray, 4.1A	None
8	6apb.1	Fusion glycoprotein F0, Fusion glycoprotein	0.88	0.67	93.18	X-ray, 3.0 Å	NAG

⁣^∗^NAG: refers to a ligand predicted by SWISS-MODEL called 2-acetamido-2-deoxy-beta-D-glucopyranose. The predicted ligands could be suggested as natural compound and they may not represent active antiviral agents.

**Table 6:** Illustration of SWISS-MODEL parameters for each built model. The generated models were selected based on the matched template and the statistical parameters of the SWISS-MODEL server.

Model	SMTL ID	Template name	GMQE	QMEANDisCo	QMEAN Z-score
1	7uja.1	A RSV variant (construct pXCS847A) F1	0.76	0.71	−3.03
2	5j3d.1	F fusion glycoprotein—crystal structure of human fab 14N4 in complex with postfusion RSV F	0.73	0.70	−0.76
3	5j3d.1	F fusion glycoprotein F0	0.73	0.70	−0.76
4	5j3d.1	F fusion glycoprotein F0	0.73	0.70	−0.90
5	7uj3.1	B RSV variant (construct pXCS847A) F1	0.80	0.79	−2.16
6	7uja.1	A RSV variant (construct pXCS847A) F1	0.76	0.71	−3.03
7	5j3d.1	F fusion glycoprotein F0	0.73	0.70	−0.90
8	6apb.1	Fusion glycoprotein F0, Fusion glycoprotein	0.74	0.71	−0.88

**Table 7:** Details of model's parameters and their templates using the I-TASSER modeling web tool (https://zhanggroup.org/I-TASSER/).

NO	PDB ID	Template name	Identity	Coverage	Normalized Z-score
1	3rkiA	Structural basis for immunization with postfusion RSV F to elicit high neutralizing antibody titers	0.93	0.99	3.11
2	3rkiA	Structural basis for immunization with postfusion RSV F to elicit high neutralizing antibody titers	0.92	0.98	3.11
3	8PHI	Crystal structure of prefusion-stabilized RSV F variant DS-Cav1	0.93	1.0	2.72
4	8T7A	Cryo-EM structure of RSV preF in complex with fab 2.4K	0.93	1.0	2.52
5	8T7A	Cryo-EM structure of RSV preF in complex with fab 2.4K	0.92	1.0	2.50

**Table 8:** Statistical parameters for the most relevant model generated by I-TASSER.

Model	Template	C-score	TM-score	RMSD (Å)
1	3rkiA	−0.70	0.62 + 14	**4.4**
2	3rkiA	0.26	0.75 + 0.10	**3.6**
3	8T7A	0.36	0.76 + 0.10	**3.5**
4	8T7A	−1.78	0.79 + 0.09	**3.2**
5	8t7A	−1.32	0.77 + 0.10	**3.4**
6	8PHI	−2.27	0.78 + 0.10	**3.4**
7	3rkiA	0.47	0.78 + 0.10	**3.4**

*Note:* The RMSD refer to the root-mean-square deviation that is used to measure the degree of the structure similarity between two proteins. Low RMSD indicates having good quality of protein model. This parameter has been used in this work to assess the quality structure of the predicted fusion protein models. Models with low RMSD values have good quality structure.

## Data Availability

The data that support the findings of this study are available from the corresponding author upon reasonable request.
